# Analysis of disease resistance of *ZmERS4* -overexpressing rice

**DOI:** 10.1371/journal.pone.0325062

**Published:** 2025-07-01

**Authors:** Wanyuan Huang, Jun Ning, Bo Su, Wenjie Liu, Ting Ding

**Affiliations:** School of Plant Protection, Key Laboratory of Biology and Sustainable Management of Plant Diseases and Pests of Anhui Higher Education Institutes, Anhui Agricultural University, Hefei, China; Osmania University, INDIA

## Abstract

The discovery of the novel genes with disease resistance and the cultivation of new varieties of maize was considered as the most economical and efficient strategy for the disease stress. In previous studies, our research team had screened and obtained an ethylene receptor protein gene *Zea mays Ethylene Response Sensor4* (*ZmERS4*) from the maize leaf, then *ZmERS4* was overexpressed in rice, followed by the obtaining of the T3 homozygous transgenic rice. Besides, the disease resistance of the *ZmERS4* -overexpressing rice was analyzed during the research, which revealed that the overexpression of *ZmERS4* in rice could enhance the resistance to *Xanthomonas oryzae* pv*. oryzicola*(*Xoc*) by reducing the expression of the sugar transporter genes and activating the expression of salicylic acid (SA) signaling-related genes at 24h post-inoculation with *Xanthomonas oryzae* pv. *oryzicola.* Besides, an increasing trend could be observed in the hydrogen peroxide content, which could be attributed to the overexpression of *ZmERS4*. Furthermore, it was indicated by high-performance liquid chromatography-tandem mass (HPLC-MS/MS) spectrometry that the SA contents of *ZmERS4*-overexpressing rice exhibited a significant increasing trend after the pathogen infection. Nevertheless, the improved resistance of *ZmERS4*-overexpressing rice was relatively inhibited after the pretreatment of the SA biosynthetic inhibitor, 1-amino-benzotriazole (ABT). According to the above results, it was assumed that *ZmERS4* exhibited significant functions during the regulation of rice resistance to *Xanthomonas oryzae* pv. *oryzicola*, and the regulatory functions were mainly based on the inducement of plant oxidative burst activity and activation of the SA signaling pathway. Overall, these findings could provide genetic resources and a data basis for the exploration and evaluation of valuable disease-resistant genes from maize.

## Introduction

In recent years, diseases have gradually become an important factor that restricted the high and stable yield of maize, which could be attributed to the adjustments of cropping systems, transformation of cultivation methods, the change of corn varieties, and global warming. For instance, the southern corn rust was prevalent in the Huang-Huai-Hai region in China in 2015 [1]. Corn stalk rot exhibited an epidemic trend in the northwest region in 2016 and in the Huang-Huai-Hai region in 2017 in China [2–3]. Based on the practice and research during the past years, the exploration of the novel disease-resistant genes and the cultivation of new maize varieties were found to be the most economical and effective strategy to deal with the disease stress.

Ethylene receptors were referred to as the initial components in the ethylene signaling pathway, which could be bound to the ethylene and activate the downstream ethylene signaling pathway, and they exhibited crucial functions in the synthesis pathway and signal transduction of ethylene. To date, arious ethylene receptor proteins in different species have been identified, such as *Ethylene Response 1* (*ETR1*), *Ethylene Response 2* (*ETR2*), *Ethylene Insensitive 4* (*EIN4*), *Ethylene Response Sensor 1* (*ERS1*), and *Ethylene Response Sensor 2* (*ERS2*) in *Arabidopsis* [4–7], six ethylene receptors in tomato [8–12] and five ethylene receptors in rice [13–14]. Furthermore, functional studies have been carried out on mutants or overexpressing plants of the above ethylene receptors. Zhou *et al.* [15] cloned the *Cucumis melo Ethylene Receptor2* (*CmETR2*) from *Chrysanthemum* and transferred it into *Arabidopsis*, and it was found that the overexpression of *CmETR2* could delay the flowering of *Arabidopsis*. Additionally, it was discovered by Huang *et al.* [16] that the expression of *Oryza sativa Ethylene Receptor4* (*OsETR4*) in rice exhibited an increasing trend during the seed germination and seedling stages under the condition of low-temperature stress. In addition, the disease resistance of three ethylene receptor mutants of rice, which were inoculated with *Rhizoctonia solani* for 20 days, appeared to be significantly lower versus the wild-type controls, which could indicate that the loss of ethylene receptors would affect the resistance of rice to sheath blight [17]. Therefore, ethylene receptors were confirmed to exhibit crucial functions in rice response to both biotic and abiotic stresses.

An ethylene receptor protein gene *ZmERS4* from the maize leaf has been screened and obtained by our research group, and it was found that the *ZmERS4* exhibited an upregulated trend after the *Pantoea stewartii* infection [18], which could suggest its potential involvement in plant disease resistance. Based on the advantages of the rice as a monocot model species, including an optimized genetic transformation platform and shorter experimental timelines [19], *ZmERS4* were overexpressed in rice, alongside the successful obtaining of T3 generation lines of *ZmERS4*-overexpressing rice. Given that *ZmERS4* exhibiting response to pathogenic bacterial infection, and *Xanthomonas oryzae* pv. *oryzicola* was referred to as one of the quarantine pests prohibited from entry in China [20], which was also considered as the causative agent of rice bacterial leaf streak, thus posing a major threat to the productivity of global rice. This study aimed to clarify the biological function of *ZmERS4* in response to *Xoc* infection by analyzing the disease-resistant phenotype of the transgenic rice lines, alongside the determination of the expression of the disease-resistant genes. Additionally, the signal pathway of *ZmERS4* that regulated the rice hormone response to pathogen infection was elucidated by the high performance liquid chromatography. Overall, this research could offer a new insight into the subsequent creation of *ZmERS4* overexpressing and knockout the transgenic maize lines; simultaneously, it could provide genetic resources for the mining and evaluation of superior disease-resistant genes of maize.

## Results

### Agronomic trait statistics of *ZmERS4*-overexpressing rice

The mixed leaf samples from three-leaf-stage seedlings of *ZmERS4*-overexpressing lines were collected for the extraction of DNA. According to the PCR results, the DNA of transgenic lines could amplify *GUS*(650 bp) and *ZmERS4* (1905 bp) fragments, while the wild-type plants were not detected during the test (S1 Fig). Additionally, it was found by the qRT-PCR analysis of *ZmERS4* expression in the transgenic rice lines L5, L7, and L11 that the *ZmERS4* expression appeared to be significantly higher in lines L5 and L7 versus L11 (S1 Fig). Besides, it could be indicated by the above results that the lines L5, L7, and L11 were all referred to as *ZmERS4-*overexpressing plants, and these transgenic lines would be employed for the analysis of the agronomic traits in subsequent experiments.

**Fig 1:**
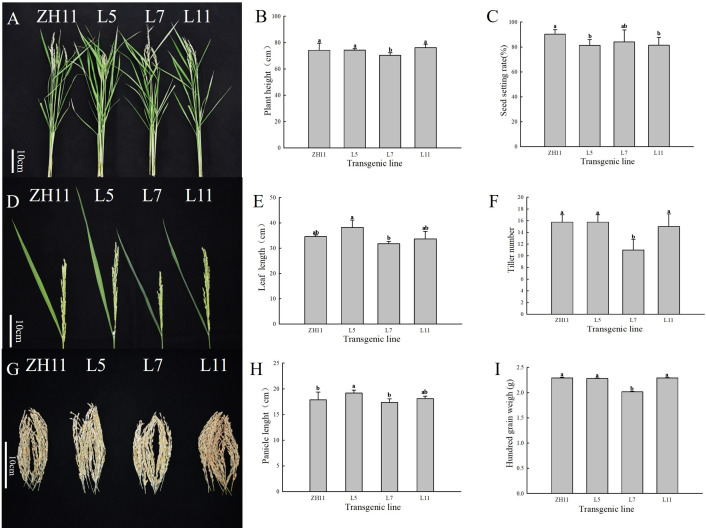
Statistics of agronomic traits of T3 transgenic lines. **(A)** The phenotype of the T3 transgenic lines; **(B)** Plant height of the T3 transgenic lines; **(C)** Seed setting rates of the T3 transgenic lines; **(D)** Phenotypic characterization of flag leaves in the T3 transgenic lines; **(E)** The flag leaf length of the T3 transgenic lines; **(F)** Tiller number of the T3 transgenic lines; **(G)** The phenotype of panicle in the T3 transgenic lines; **(H)** Panicle length of the T3 transgenic lines; **(I)** Hundred-grain weight of the T3 transgenic lines.

The height, flag leaf length, number of effective tillers, panicle length, seed setting rate, and hundred-grain weight of the transgenic lines were analyzed to investigate the actual effects of overexpression of *ZmERS4* in rice on agronomic traits. According to the results, no significant difference was observed in plant height between *ZmERS4* -overexpressing lines and wild-type rice, with an average height of around 75 cm (Fig 1A and 1B). Additionally, the flag leaf lengths of the *ZmERS4*-overexpressing lines L5, L7, L11 were 37.98, 33.79, and 34.20 cm, respectively, which were relatively longer than that of the wild plant (30.35 cm) (Fig 1D and 1E). Besides, the tiller number exhibited direct effects on yield, and it was recorded as 15–16 tillers per plant in lines L5 and L11, which was similar to wild-type, while line L7 showed significantly fewer tillers with around 10–11 tillers per plant (Fig 1F). Meanwhile, the panicle length of line L5 was approximately 20 cm, which was relatively longer than other lines (Fig 1G and 1H). Additionally, the seed setting rates of the three transgenic lines were all lower than that of the wild-type (Fig 1C). Regarding the hundred-grain weight, the line L7 showed an evidently descent (Fig 1I) compared with those of the lines L5, L11 and the wild. Overall, it was revealed that there were certain differences in agronomic traits between the *ZmERS4*-overexpressing lines and wild rice. Furthermore, it was found that transgenic lines L5 and L7 exhibited no significant difference in *ZmERS4* expression levels (S1 Fig) while they displayed distinct phenotypic variations. Given the consistent environmental conditions (light, temperature, and humidity) and cultivation management of the transgenic lines and wild plants, it was hypothesized that the differences in agronomic traits between transgenic lines L5 and L7 could be attributed to the random T-DNA insertion sites.

### Resistance analysis of *ZmERS4*-overexpressing rice to bacterial leaf streak

**Fig 2:**
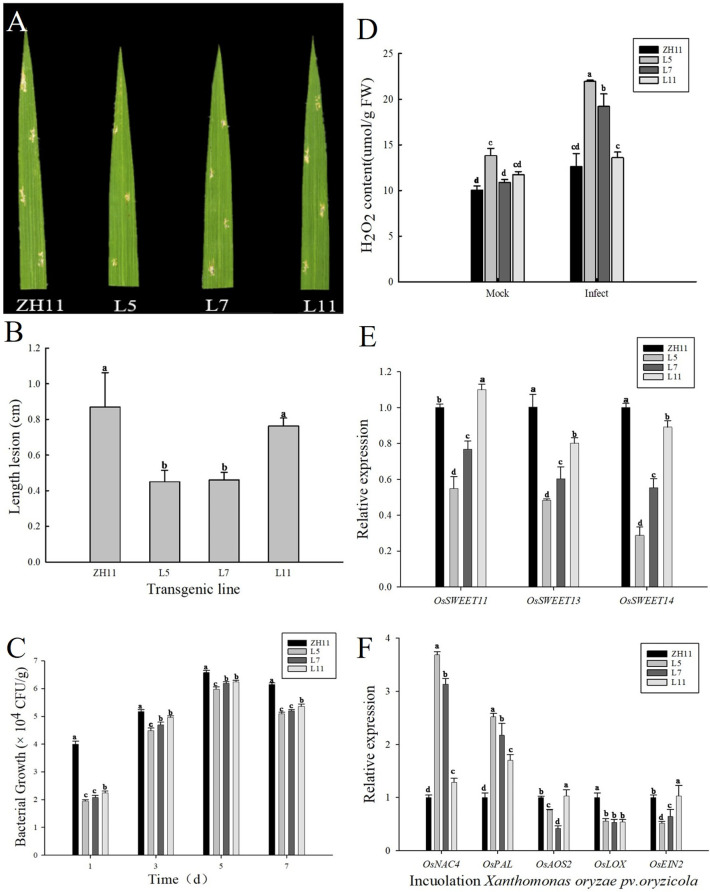
The overexpression of *ZmERS4* in rice enhanced plant resistance to *Xoc.* **(A)** Phenotypes of the different groups treated with *Xoc* at 7 d;(B) The lesion lengths of groups at 7 d after inoculation with the *Xoc*; **(C)** The population density of bacterium of different groups; **(D)** H_2_O_2_ content of different groups at 3 d after inoculation with the *Xoc*; **(E)** The expression of *OsSWEET* of the different groups treated with *Xoc* at 24 h; **(F)** The expression levels of target plant genes of the salicylic acid, jasmonic acid or ethephon pathway in different groups treated with *Xoc* at 24 h, *OsActin* and *OsGADPH* were used as reference genes for normalization.

Subsequently, the disease resistance function of transgenic lines was analyzed on the basis of clarifying the agronomic traits of *ZmERS4* -overexpressing rices. Yellow necrotic spots were observed on plant leaves from different groups 3 days after inoculation with the *Xanthomonas oryzae* pv. *oryzicola*, and the necrotic spots gradually expanded with period after inoculation (Fig 2A). Besides, the lesion lengths of different groups were measured on the 7th day after inoculation with *Xoc*. and it was found that the lesion lengths of the transgenic lines L5 and L7 were 0.45 and 0.46 cm, which exhibited an obvious decreasing trend versus the wild-type (0.87 cm). However, no significant difference was observed in the transgenic line L11 (0.76 cm) and wild-type (0.87 cm) (Fig 2B). Overall, the above results could indicate that the expression of *ZmERS4* in rice exhibited the ability to improve the plant resistance to *Xoc*.

The bacterial populations in plant leaves of different groups at 1,3, 5, and 7 d after pathogen inoculation were tested in this study for the exploration of the connection between the bacterial populations in transgenic rice and disease resistance. According to the findings, it could be indicated that the bacterial populations in transgenic lines were consistently lower than those in wild plants at different time points. On the 7th day post-inoculation, the bacterial count in transgenic lines L5, L7 and L11 remained at 5.07 × 10^4^, 5.23 × 10^4^ and 5.41 × 10^4^ CFU/g, respectively, while that of the wild-type was 6.24 × 10^4^ CFU/g (Fig 2C), which suggested the enhancing effects of the *ZmERS4*-overexpressing in rice on the plant resistance to *Xoc*.

Subsequently, the hydrogen peroxide contents in plants before and afte with *Xoc* infection on the 3rd were measured, and it was found that the H_2_O_2_ in different groups all exhibited a rapidly accumulative trend after the infection with *Xoc*, and the H_2_O_2_ contents in transgenic L5, L7 and L11 reached 21.95µmol/g, 19.23µmol/g and 13.62 µmol/g, respectively, which were relatively higher versus the wild-type (12.63µmol/g) (Fig 2D), revealing the induction of the *ZmERS4*-overexpressing in rice on the accumulation of H_2_O_2_ against *Xoc* infection.

Sugar transporters tended to participate in various plant physiological processes, including plant development and plant-pathogen interactions. Additionally, the expression of various genes that were involved in sugar transport was assessed in different transgenic lines at 24 h post-inoculation with *Xoc*, which were composed of *Oryza sativa Sugars Will Eventually Be Exported Transporter 11* (*OsSWEET11*), *Oryza sativa Sugars Will Eventually Be Exported Transporter 13* (*OsSWEET13*), and *Oryza sativa Sugars Will Eventually Be Exported Transporter 14* (*OsSWEET14*) genes. And it was found that the level of *OsSWEET11*, *OsSWEET13,* and *OsSWEET14* in transgenic lines L5 and L7 appeared to be significantly lower versus the wild ZH11 (Fig 2E). Regarding the transgenic line L11, the level of these genes was obviously higher than that of lines L5 and L7, and the *OsSWEET11* expression was notably upregulated compared to the wild group (Fig 2E). Besides, the downregulation of *SWEET* genes was confirmed to enhance the plant disease resistance according to the previous reports, and it was speculated that the transgenic lines L5 and L7 exhibited better resistance to bacterial leaf streak.

Additionally, the predominant signaling genes related to SA, JA, and ET disease resistance pathways were assessed in transgenic lines, such as *Oryza sativa NAC domain-containing protein 4* (*OsNAC4*), *Oryza sativa Phenylalanine ammonia-lyase* (*OsPAL*), *Oryza sativa Lipoxygenase* (*OsLOX*), *Oryza sativa Allene oxide synthase 2* (*OsAOS2*), and *Oryza sativa Ethylene Insensitive 2* (*OsEIN2*). At 24h post-inoculation with *Xoc*, the level of SA-related genes *OsNAC4* and *OsPAL* was generally elevated in transgenic lines L5, L7, and L11 versus the wild-type. Notably, the expression of *OsNAC4* in L5, L7, and L11 were 3.69, 3.13, and 1.29 times, which were higher versus the wild-type control, respectively. Additionally, the expression levels of *OsPAL* were 2.52, 2.17, and 1.70 times higher in these lines, respectively (Fig 2F). However, compared with the wild-type control, the expression of JA-related genes *OsLOX*, *OsAOS2,* and ET-related gene *OsEIN2* were not induced in transgenic lines. Therefore, it was inferred that the overexpression of *ZmERS4* in rice might exhibit enhancing effects on the resistance to bacterial leaf streak based on the activation of the SA-dependent signaling pathway, which contributed to the plant defense responses.

### *ZmERS4*-overexpressing rice exhibited resistance to *Xoc* through the SA-mediated signaling pathway

**Fig 3:**
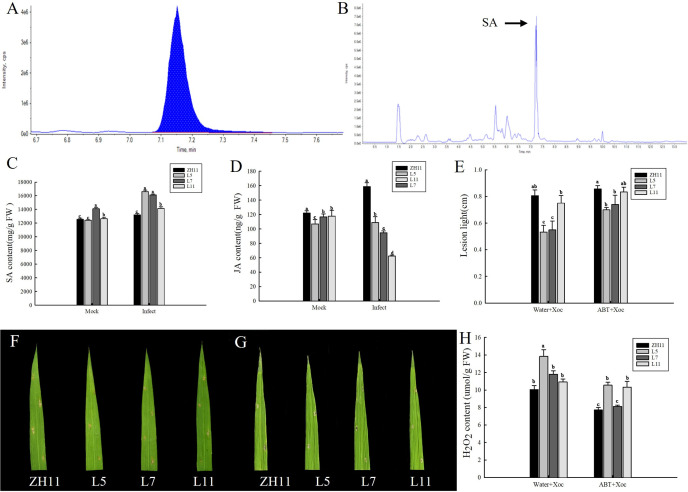
The overexpression of *ZmERS4* in rice enhanced resistance to *Xoc* through the SA mediated signaling pathway. **(A)** SA chromatogram of standard SA; **(B)** SA chromatogram of transgenic line L7 infected with *Xoc*; (C) and **(D)** Salicylic acid and Jasmonic acid contents in the different groups at 24 h post- *Xoc* infection; **(E)** Lesion lengths of the different groups; **(F)** Phenotypes of the different groups at 7 d post- *Xoc* without ABT treatment; **(G)**Phenotypes of the different groups at 7 d post- *Xoc* infection, which were pretreated with ABT for 24 h; **(H)** Detection of H_2_O_2_ content of rice leaves at the 3 rd in different groups.

The involvement of *ZmERS4* in plant defense mechanisms against *Xoc*It was confirmed by the above results, but the dynamic content changes of endogenous SA in transgenic lines before and after pathogen infection remained unclarified. The HPLC-MS/MS was employed in the evaluation of the SA contents in transgenic lines L5, L7, and L11. According to the results, the retention time of the peaks of the SA extracts corresponded to the standard SA, which was at 7.18 ± 0.03 min (Fig 3A and 3B). Additionally, no obvious difference could be found between the transgenic SA contents and the wild-type plants before the *Xoc* infection. Notably, the SA contents of transgenic L5, L7, and L11 accumulated rapidly after the *Xoc* infection, which reached 16.62, 16.13, and 14.16 µg/g FW at 24 h, respectively, and these values were higher versus the wild-type (13.24 µg/g FW) (Fig 3C). Within the aspects of plants, the SA and JA were two opposing signaling pathways that were employed by plants to resist the pathogen invasion. Therefore, the JA content in different groups was also detected, and it was found that the JA content in all transgenic lines exhibited a significant decreasing trend versus the lines before *Xoc* inoculation (Fig 3D). Overall, it could be indicated by these results that the overexpression of *ZmERS4* in rice could induce the accumulation of SA and inhibit the formation of JA, thus contributing ot the activation of the SA signaling pathway against *Xoc*.

Various groups were pretreated by 0.1 mmol/L 1-amino-1,2,4-triazole (ABT) (a SA synthesis inhibitor) for 24 h, which was aimed to further explore the mechanisms by which the *ZmERS4* enhance the resistance through the SA signaling pathway. Subsequently, the plants were inoculated with the *Xoc*, and the lesion lengths of the groups were measured on the 7th day post-inoculation. Additionally, more severe symptoms were observed in the *ZmERS4*-overexpressing lines and wild-type plants (pretreated with ABT) after the inoculation (Fig 3F and 3G), and the lesion lengths of lines L5, L7, L11 and wild ZH11were 0.70, 0.74,0.83 and 0.80 cm, respectively, while the negative groups were 0.53, 0.55,0.75, and 0.80, respectively (Fig 3E). Furthermore, the H_2_O_2_ contents in the ABT-pretreated transgenic lines L5, L7, and L11 were 10.56, 8.13 and 9.33µmol/g, which were significantly lower versus the negative groups L5(13.85µmol/g), L7(11.80µmol/g), and L11(10.92µmol/g) (Fig 3H). It was indicated by the results that the ABT application could reduce the *ZmERS4*-overexpressing rice resistance to *Xoc*, conversely suggesting the enhancing effects of the overexpression of *ZmERS4* in rice on resistance to *Xoc*.

## Discussion

Ethylene receptors were considered as signaling factors, which exhibited crucial functions in ethylene biosynthesis and signal transduction pathways, and they showed the ability to determine the sensitivity of plants to ethylene, control the processes of plant maturation and senescence, and mediate the responses to environment [21–23]. It was found in previous studies that the mutants or overexpression of the ethylene receptor in plants could regulate the expression of defense genes, participate in pathogen-induced cell death alongside the alteration or disruption of the ethylene biosynthesis and signal transduction processes in host plants, thereby exhibiting either disease resistance or susceptibility. Additionally, the number of pathogen in *Arabidopsis* etr1−1knockout mutants was relatively lower than that in wild-type plants after the infection with *Verticillium dahliae*, and resistance genes such as *Chitinase 1* (*CHI-1*), *Chitinase 2* (*CHI-2*), *Pathogenesis-related protein 1* (*PR1*), *Pathogenesis-related protein 2 (PR2*), and *Pathogenesis-related protein 5* (*PR5*), along with GST cascade signal pathway genes like *Glutathione S-transferase F12* (*GSTF12*) and *Glutathione S-transferase U16* (*GSTU16*), and MYB transcription factor *MYB domain-containing protein 75* (*MYB75*) were significantly upregulated, which could demonstrate the stronger disease resistance [24]. Subsequently, *ETR1* was found by Pantelides et al. to participated in the infection of *Fusarium oxysporum*. Compared with the wild type, the *etr1−1* mutant showed a mild symptom and upregulated expression of *PR1*, *PR2*, and *PR5* resistance genes [25]. Besides, a series of disease resistance analyses were carried out on *ZmERS4* -overexpressing rice, and it was found that the overexpression of *ZmERS4* in rice could improve the resistance to hemibiotrophic *Xoc* by inducing hydrogen peroxide (H_2_O_2_) accumulation, reducing the expression of sugar transporter-related genes, and participating in the regulation of salicylic acid hormone signaling, which could in turn enhance the resistance to bacterial leaf streak. Notably, the above findings were in agreement with the previous studies, which could verify the crucial functions of the ethylene receptors in disease resistance based on the modulation of the plant hormone signaling. In future research, transcriptomics, metabolomics, and weighted gene co-expression network analysis would be employed to establish the signal network of *ZmERS4* regulating plant hormone response to pathogen infection.

In addition, the effects of the expression of ethylene receptors in plants or the absence of ethylene receptors in plants on their growth were also investigated. The overexpression of *OsETR4* in the rice was confirmed to improve the germination rate and seeding growth of the rice under low-temperature stress [16]. Additionally, Liu et al. [26] found the mutation of *ERS1* in Arabidopsis ethylene receptor mutant, which contained *ETR1*, could alleviate the constitutive ethylene response phenotype, while overexpression of the *ERS1* enhanced the inhibiting effects of plant growth, which could suggest the dual functions of *ERS1* in the regulation of ethylene responses. In this study, overexpression of *ZmERS4* in rice was found to enhance the plant disease resistance, alongside the further alteration of some agronomic traits such as tiller number, seed setting rate, and hundred-grain weight, which were lower versus the wild-type rice. Additionally, previous studies revealed that *ZmERS4* was localized on the plasma membrane and belonged to the first subfamily of ethylene receptor proteins [18], and it was indicated by previous research that *ERS1* of the first subfamily of ethylene receptor proteins could bind to *ETR1* for the formation of a heterologous complex, which could in turn reduce the activity of *ETR1*. Moreover, the overexpression of *ERS1* could inhibit the function of *ETR1*, thereby enhancing the ethylene response [26]. Based on the above findings, it was speculated that the overexpression of *ZmERS4* in rice could enhance the binding between *ZmERS4* with ethylene, which could in turn partially suppress the plant growth, resulting in a decreasing trend in traits, such as effective tiller number and seed setting rate.

In recent years, the ethylene signaling pathway has been confirmed to have extensive cross-talk with other hormone pathways such as JA, SA, and auxin, and these pathways were found to collectively regulate plant growth and responses to biotic and abiotic stress [27]. For instance, the regulatory networks of ethylene and auxin were interconnected, with both hormones pathway converging on the core regulatory pathway for root hair development through *Root Hair Specific 4* (*RSL4*) [28–29]. Additionally, the *Ethylene Responsive Factor 1* (*ERF1*) could participate in the ethylene-mediated defense responses, alongside the regulation of JA-mediated defense responses [30]. Additionally, Li et al. [31] revealed that the SA signaling in Arabidopsis was repressed by JA and ethylene under both normal and low temperatures. Furthermore, Wang et al. [32] reported the synergistic effects of ethylene and SA on the promotion of leaf senescence in Arabidopsis by *Ethylene Insensitive 3* (*EIN3*), which was referred as a crucial transcription factor of ethylene signaling, and it would interact with the core SA signaling regulator *Nonexpressor of Pathogenesis-Related Genes 1* (*NPR1*) in senescing leaves. Besides, the team found that the overexpression of the *ZmERS4* gene in rice exhibited enhancing effects on the disease resistance to bacterial leaf streak based on the activation of the SA signaling pathway. According to previous reports [18], it was speculated that the expression of the ethylene receptor *ZmERS4* in rice could enhance the upregulation of downstream target genes in the ethylene signaling pathway, and the upregulation could subsequently trigger the interactions between the target genes and key proteins in the salicylic acid (SA) pathway. Additionally, such interactions might induce the transcriptional activation of SA-responsive pathogenesis-related genes, which could ultimately result in the activation of the SA signaling pathway. In future research, it was planned to analyze the expression of downstream transcription factors in the ethylene signaling pathway within the transgenic rice infected with pathogens, alongside the screening for proteins in the salicylic acid pathway that might interact with transcription factors in the ethylene signaling pathway. Overall, the results would refine the functional characterization of *ZmERS4*.

## Materials and methods

### Pathogen cultivation

*Xanthomonas oryzae* pv. *oryzicola* (*Xoc*) was provided by the Anhui Province Key Laboratory of Integrated Pest Management on Crops, College of Plant Protection, Anhui Agricultural University, Hefei, China, and the samples were grown at 28°C for 36 h in an NA liquid medium.

### Plant materials

The wild-type rice used in this experiment was Zhonghua 11 transgenic lines were all derived from the same parental Zhonghua 11 background. And the wild-type and transgenic lines were grown under the condition of 28°C with a 16/8 h light/dark photoperiod, alongside a 60–70% relative humidity.

### Detection of *ZmERS4* transgenic plants

During the three-leaf-one-heart stage, transgenic T3 rice lines (L5, L7, L11) were selected for DNA extraction by the CTAB method. Additionally, the primers were determined according to the CDS length of the *ZmERS4* gene and the *GUS* gene on the vector (Table 1). Besides, PCR was performed to identify the positive transgenic plants. Each PCR tube contained 12.5 μL of 2 × Rapid Taq Master Mix, 1 μL cDNA, 1 μL of each primer, and 9.5 μL ddH_2_O. The thermal cycling conditions were as follows: 10 min at 95°C, followed by 35 cycles of 15 s at 95°C, 15 s at 55°C, and 30 s at 72°C.

**Table 1:** List of PCR and primer sequences.

Primer names	Primer sequence (5ˊ-3ˊ)
*GUS-F*	GCGAAGTCTTTATACCGAAAGGTTG
*GUS-R*	GCCCTTCACTGCCACTGACC
*qZmERS4-F*	AAGCTCGGCGTGTGTGACAA
*qZmERS4-R*	CTGCACTGCTTGGCCAGACT
*qOsActin-F*	GACCTTGCTGGGCGTGAT
*qOsActin-R*	GTCATAGTCCAGGGCGATGT
*qOsGADPH-F*	CTTCGGCATTGTTGAGGGTTTG
*qOsGADPH-R*	TCCTTGGCTGAGGGTCCGTC

Subsequently, RNA from the transgenic T3 rice lines (L5, L7, L11) was employed as a template for the evaluation of the *ZmERS4* expression, in which the β-Actin served as the reference gene, and the primer sequences were listed in Table 1. Besides, the experimental conditions were: 30 s at 94°C, 40 cycles for 5 s at 94°C, and 15 s at 55°C. Moreover, the 2^-ΔΔCt^ method was employed for the analysis of the data.

### Agronomic trait investigation of *ZmERS4* transgenic T3 lines

The transgenic lines L5, L7, L11 (T3 generation), and wild-type Zhonghua 11 were planted in the teaching and practising base of Anhui Agricultural University (117.2570 E, 31.8619 N) in May 2022 for the exploration of the functions of the heterologous overexpression of *ZmERS4* on rice growth and development. Additionally, the experiment followed a completely randomized design, with 15 plants in one plot, five plants from each plot were randomly selected to measure plant height, panicle length, flag leaf length, tiller number, seed setting rate, and hundred-grain weight [33].

### Disease resistance analysis of *ZmERS4* transgenic rice

Seeds of transgenic rice lines L5, L7, L11 (T3 generation), and wild-type Zhonghua 11 were soaked in water under the room temperature for 24 h, followed by germinating in petri dishes. During the one-leaf-one-heart stage, the seedlings were transplanted to the plastic boxes (51 cm × 38 cm × 25 cm) with 20 plants per box and grown hydroponically in a greenhouse.

During the jointing stage of the seedlings, leaves of different treatments were inoculated with *Xoc* suspension (OD_600_ = 0.5) by the needle-pricking method, and then the samples were maintained under 85% humidity for 18 h. Besides, the plants treated with sterile NA liquid medium were used as the negative controls.

A single treatment was composed of 9 plants, which could be assigned to three replicates., Additionally, the length of disease lesions of each treatment was measured 7 days after the post-inoculation. Additionally, bacterial numbers within the mesophyll tissues were estimated by the serial dilution coating method [34] 1, 3, 5, and 7 days after pathogen infection, with three replicates in each group (three plants per replicate).

The wild-type and transgenic rice were treated with 0.1 mmol/L 1-amino-benzotriazole (ABT) to verify the actual effects of the overexpression of *ZmERS4*. Additionally, the plants processed by sterile distilled water containing 0.02% Tween-20 were employed as the negative controls. Subsequently, the plants were inoculated with *Xoc* after 24 h*,* and the length of disease lesions of the groups was measured at 7 days post-inoculation.

### Quantitative PCR

The rice leaves were obtained for RNA extraction with TRIzol reagent 24 h after infection. Additionally, the qRT-PCR was operated by AceQ qPCR SYBR Green Master Mix (Vazyme Biotech Co., Ltd., Nanjing, China), and 10 μL AceQ qPCR SYBR Green Master Mix, 2 μL cDNA, 1 μL of each primer (Table 2), and 6 μL RNase-free Water were incorporated in each PCR tube. The thermal cycling conditions were as follows: 5 min at 95°C, 40 cycles for 10 s at 95°C, and 30 s at 60°C. Additionally, the analysis of the data was according to the previous studies [35]. Besides, all the samples were assigned to three replicates for each experiment.

**Table 2:** Primer sequences for fluorescence quantification genes.

Primer names	Primer sequence (5ˊ-3ˊ)
*OsActin-F*	GACCTTGCTGGGCGTGAT
*OsActin-R*	GTCATAGTCCAGGGCGATGT
*OsGADPH-F*	AAGCCAGCATCCTATGATCAGATT
*OsGADPH -R*	CGTAACCCAGAATACCCTTGAGTTT
*OsSWEET11-F*	CAAGAAGAAGTCGACGGGAG
*OsSWEET11-R*	CTCGAGTTGGTCTTCACCAG
*OsSWEET13-F*	TGTGCAGTACCACGTGCC
*OsSWEET13-R*	AATTAATTGGTGGTAGCTTGTCTC
*OsSWEET14-F*	AAGGATAGATGCATATGTGTTCGT
*OsSWEET14-R*	GCATGCATAAAATGCGACGG
*OsPAL-F*	GCACATCTTGGAGGGAAGCT
*OsPAL-R*	GCGCGGATAACCTCAATTTG
*OsNAC4-F*	AGACGGACTGGATCATGCACGA
*OsNAC4-R*	TCCAGCTTCTGTGAGCCCTTCTTG
*OsLOX-F*	GCATCCCCAACAGCACATC
*OsLOX-R*	AATAAAGATTTGGGAGTGACATATTGG
*OsAOS2-F*	CTCGTCGGAAGGCTGTTGCT
*OsAOS2-R*	ACGATTGACGGCGGAGGTT
*OsEIN2-F*	CAAGGAACCAGTGACAACCA
*OsEIN2-R*	GCAGTCGTCTCCGCAGTTAG

### H_2_O_2_ detection

The leaves were all harvested at 3 days post-inoculation for the detection of the H_2_O_2_ by the Keming kit (Suzhou Keming Biotechnology Co., Ltd., Suzhou, China). Relying on the formation of an orange complex by H_2_O_2_ and titanium ions in an acidic medium, the titanium sulfate spectrophotometric method was employed in the assessment of the H_2_O_2_ concentration in transgenic lines, and he wild-type Zhonghua 11 was employed as the negative control. Three replicates were operated in each group, with each replicate consisting of a mixture of three rice leaves.

### Detection of SA in *ZmERS4*-overexpressing rice

The SA contents before and after 24 h infection with *Xoc* were monitored by an Agilent 1290 HPLC system coupled with an Applied Biosystems 6500 quadrupole MS/MS mass spectrometer, which was run in multiple reaction monitoring mode (Nanjing Convinced-Test Technology Co., Ltd., Nanjing, China). Additionally, the isopropanol-water-hydrochloric acid extraction method was employed in the extraction of SA. Besides, the samples were eluted with a gradient, which applied 0.1% formic acid in methanol (A) and 0.1% acetic acid in water (B) as mobile phases. Three replicates were operated for each group, with each replicate containing three plants.

### Data analysis

The statistical data was processed by SPSS, and the statistical differences (p < 0.05) were evaluated by one-way analysis of variance.

## Supporting information

S1 Fig Identification of positive seedlings in *ZmERS4*-Overexpressing rice. (A) PCR analysis of *ZmERS4*. M: 2K DNA marker; (B) PCR analysis of *GUS*. M: 2K DNA marker; (C) qRT-PCR analysis of *ZmERS4* expression in T3 transgenic rice lines.
(TIF)
