# Supplementary figures and images for: Analysis of disease resistance of ZmERS4 -overexpressing rice
Source: PLoS One. 2025 Jul 1;20(7):e0325062. doi: 10.1371/journal.pone.0325062 (PMC12212565; doi:10.1371/journal.pone.0325062)

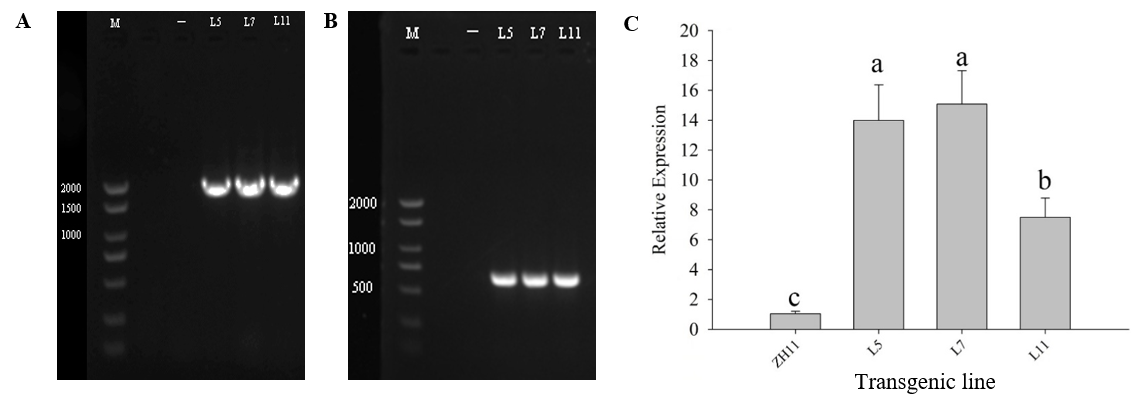

Supplement: S1 Fig — (A) PCR analysis of ZmERS4. M: 2K DNA marker; (B) PCR analysis of GUS. M: 2K DNA marker; (C) qRT-PCR analysis of ZmERS4 expression in T3 transgenic rice lines. (TIF) [file pone.0325062.s001.tif]
